# Improving comprehensive geriatric assessments with the clinical frailty scale: a focus group study

**DOI:** 10.1186/s12877-025-05987-6

**Published:** 2025-05-08

**Authors:** Synneve Dahlin-Ivanoff, Frida Mjörnstedt Oleander, Katarina Wilhelmson

**Affiliations:** 1 Institute of Neuroscience and Physiology, Department of Psychiatry and Neurochemistry, Sahlgrenska Academy, University of Gothenburg, Gothenburg, Sweden; 2 Centre for Ageing and Health – AgeCap, University of Gothenburg, Gothenburg, Sweden; 3 Region Västra Götaland, Department of Acute Medicine and Geriatrics, Sahlgrenska University Hospital, Gothenburg, 413 45 Sweden; 4 Region Västra Götaland, Department of Geriatrics, Sahlgrenska University Hospital, Mölndal, 43180 Sweden

**Keywords:** Comprehensive geriatric assessment, Clinical frailty scale, Frail older people, Integrated care, Focus groups

## Abstract

**Background:**

The purpose of this study is to exploratively evaluate the geriatric team’s views on the implementation of the Comprehensive Geriatric Assessment (CGA) and Clinical Frailty Scale (CFS) on frail older people with acute orthopaedic disorders who are cared for in two geriatric care wards in the southwest of Sweden.

**Methods:**

A qualitative design with focus groups was applied, based on a social constructivist research tradition. This approach differs from other qualitative methods, such as interviews, in that it encourages interaction between research participants and contributes to shedding light on a collective understanding of the world. This means that the analysis is based on the group’s collective input, not individual statements. The study group consisted of 21 professionals participating in four focus groups, with five to six participants per group. The participants in each group represented different professions within the geriatric team, including nurses, nursing assistants, physicians, occupational therapists, and physiotherapists.

**Results:**

The results underscore the importance of the CFS as the basis for CGA, emphasizing the effectiveness of the scale as a shared instrument promoting collaboration in healthcare. Our study uniquely points out the lack of research exploring the team-based use of tools for conducting a frailty assessment using the CFS. The study also highlights the importance of effective teamwork and a person-centred approach. The balance between person-centred care and what is feasible within the organization is crucial to providing the best possible care for patients.

**Conclusions:**

In Sweden, as in other places, how healthcare staff experience their work is key to the quality of care. The study concludes that positive staff experiences with new approaches drive healthcare improvement, benefiting patients and society. This highlights the potential for further improvements in healthcare delivery through continued innovation and collaboration.

**Trial registration:**

Clinical trial number: not applicable.

## Background

The current landscape of acute care is highly specialized, yet it often falls short in meeting the comprehensive needs of frail older persons. This exposes them to avoidable risks, such as the loss of functional abilities, leading to unnecessary care needs and decreased wellbeing. Care is often fragmented, and the coordination and integration between care settings has been identified as essential for the quality of care for this frail population [1]. Effective coordination and integration require appropriate actions from caregivers when a person is transferred from one caregiver to another, thereby bridging the gap and constructing a “healthcare chain” [2].

The Comprehensive Geriatric Assessment (CGA) is a multidimensional process designed to evaluate the overall health status of older persons, particularly those who are frail. The principal domains assessed are according to Stefanacci [3] functional ability, physical health, cognition and mental health and socioenvironmental situation [3]. CGA should embody three key principles: comprehensiveness, person-centredness, and multidisciplinarity. Comprehensiveness involves healthcare providers identifying and addressing all of a patient’s experienced health needs. Person-centred care [4] prioritizes the patient’s needs, preferences, and values. It involves creating an individualized care plan that includes preventive, therapeutic, rehabilitative, and follow-up actions. The goal is to use resources optimally to achieve the highest degree of independence and improve the patient’s quality of life. Multidisciplinarity involves the participation of multiple healthcare professionals from different disciplines in the care of a patient [6]. This principle recognizes that older persons require support from various professions and that a multidisciplinary team can provide valuable, comprehensive care [5].

In in-hospital care for geriatric patients, CGA is considered the gold standard and has in many studies been confirmed to be effective [6]. Despite the benefits of the current CGA, there are still some limitations. To provide appropriate assessment for patients with multimorbidity, frailty, and complex care needs, many methods are used to identify older people’s problems, care needs, and preferences. However, limited knowledge of how older people’s problems and care needs should best be assessed using a CGA has led to the use of different instruments [7]. The Clinical Frailty Scale (CFS), first described in 2005, is a semi-quantitative tool that involves using clinical judgment to interpret and categorize observations into a scale or index, rather than relying solely on precise numerical measurements. It is used to estimate a person’s degree of frailty on a scale of 1 (very fit) to 9 (terminally ill). The CFS is a marker of biological age [8].

Stoop et al. [9] discuss the importance of person-centred care in the context of integrated care for older people. Barr et al. [10] define integrated care as a coordinated approach to healthcare that emphasizes collaboration between various healthcare professionals to provide comprehensive and continuous care to patients. This approach aims to bridge gaps between different services and sectors, ensuring that patients receive holistic and seamless care tailored to their personal needs. Stoop et al. [13] emphasize that while tools like the CFS are valuable, they should not be used in isolation, and [11] argue that person-centredness goes beyond these clinical measures. Person-centred care involves empowering and encouraging people to participate actively in decision-making processes about their own care, establishing an accommodating, cooperative, and ongoing relationship between the professional, the person receiving care, and the informal carer, and understanding the specific (health) concerns of the person. Therefore, while the CFS [12] is a useful tool, it should be part of a comprehensive assessment that also includes elements of person-centred care. This approach ensures that care and support reflect the needs and preferences of the person, leading to more positive experiences of care and support. This aligns with the broader shift in healthcare towards more holistic, person-centred models of care.

The article “Moving from Care Coordination to Care Integration” [2] discusses the transition from coordinated care to integrated care, which can lead to improved patient outcomes and experiences as well as reduced healthcare costs. While integrated care and person-centred care share the common goal of placing the patient at the centre, they do so through different mechanisms. Integrated care focuses on system-wide coordination and collaboration, while person-centred care emphasizes individualized attention and patient empowerment. The healthcare aims to ensure that frail older persons receive person-centred, continuous and integrated care across hospital, regional, and municipal settings. At every stage, the care provided is intended to be person-centred, meaning it respects and responds to the individual needs and preferences of the patient. This approach is hypothesized to safeguard the dignity of older persons by reorganizing care towards a continuum of care. This continuum is characterized by a respectful and empathic approach that acknowledges the abilities and needs of each older person. As part of a larger research programme, we aim to bridge the gap between hospital care and regional and municipal care for frail older persons through a comprehensive geriatric assessment throughout the healthcare chain. This approach ensures that the patient feels valued and empowered. By integrating the healthcare chain, the research programme aims to provide a seamless care experience for frail older persons, ensuring they receive the right care at the right time in the right place. The present study represents a first step in the development of how hospitals work with frail older people with acute orthogeriatric disorders. Therefore, the purpose of this study is to exploratively evaluate the geriatric team’s views on the implementation of the Comprehensive Geriatric Assessment (CGA) and Clinical Frailty Scale (CFS) on frail older people with acute orthopaedic disorders who are cared for in two geriatric care wards in the southwest of Sweden.

## Methods

A qualitative design with focus groups was used to generate data on staff experiences of using CGA with frail older persons [13]. The method is ideal for exploring staff experiences. Based on social constructivism, the focus group methodology builds on interaction between participants [14] to clarify their views and experiences and provide them with opportunities to stimulate each other in discussions to explore new issues that arise [14]. The knowledge that the focus group method generates is based on collective, common experiences [15] and focuses on the variation in the collective understanding that emerges from the discussion [15, 16]. The project was approved by the Ethics Review Board (Dnr 899 − 15). All research data is handled in accordance with GDPR. This study was conducted in two orthogeriatric wards at a university hospital in southwest Sweden that recently had implemented the CGA and the CFS. Two other geriatric wards at the same hospital had been using the CGA framework for several years. First, the staff underwent training to enhance their skills prior to the introduction of the new working method. All staff received explanations of the concepts of CGA, comprehensiveness, person-centredness, multidisciplinarity, and the CFS. They worked with the routine for the CGA working method and its round structure, including the distribution of responsibilities. The adoption of this new method introduced new responsibilities for the geriatric team. These included modifying the team structure to facilitate a daily meeting of approximately 30 min with all staff members in attendance. During these meetings, the team was to collectively conduct a frailty assessment using the CFS. An outsider (usually a section leader) attended the round once a week at the beginning of the project. After the round, they held a reflection session to discuss how everyone experienced the round, its structure, time efficiency, what worked well, what worked less well, and whether everyone had the opportunity to speak. They also focused on the use of the digital overview board, ensuring all essential points were covered, and considered what could be done differently. This process ensured that all staff had experience of CFS and CGA before participating in the focus groups.

### Participants

The study group consisted of 21 professionals with experience of working with CGA and the CFS, participating in four focus groups with five to six participants per group. To create dynamic focus groups, both homogeneity and heterogeneity (Dahlin-Ivanoff & Hultberg 2006) were considered when choosing participants. Heterogeneity is needed to cover diversity within the chosen target group, allowing participants to reflect upon each other’s experiences, and was considered in all focus groups. The participants in each focus group represented the different professions from the geriatric team, such as nurses, nursing assistants, physicians, occupational therapists, and physiotherapists. Additionally, a dietitian and a pharmacist participated in one group each. This diverse composition was crucial to ensure a broad representation of the target group and foster an open discussion climate [17]. While homogeneity, where group participants share similar experiences, is essential for stimulating discussion, heterogeneity – characterized by differences between group participants – contributes to the diversity within the selected target group [15]. Despite the importance of heterogeneity, the primary focus remained on homogeneity. This was achieved through shared experiences of being part of the same team and being involved in the implementation of the new working method. To experience a sense of commonality and facilitate the initiation of a discussion, it was important that participants were familiar with the topic under discussion and ideally had different perspectives.

### Procedure

The focus group discussions took place in the hospital conference room. Each session lasted no more than 1.5 h and was facilitated by an experienced moderator specialized in conducting focus groups (SDI, the first author). The moderator guided the discussions, using carefully formulated questions to elicit participants’ views and experiences. These questions also encouraged participants to engage with each other, explore new issues, and address the following key questions:

How do you experience the implementation of the Comprehensive Geriatric Assessment in your department?

What are your experiences of how the Clinical Frailty Scale affects Comprehensive Geriatric Assessment in your work in the multidisciplinary geriatric team?

How do you define person-centred care, and how is it applied in your work?

What does teamwork mean to you, and how does it impact patient care?

The discussion centred around experiences of working with Comprehensive Geriatric Assessment, focusing on comprehensiveness, person-centredness, multidisciplinarity, and the use of the Clinical Frailty Scale.

### Analysis

The analysis was conducted in a stepwise process inspired by the method for focus groups [13, 15]. All text was kept in Swedish until the end of the analysis procedure to stay close to the data and not lose content and meaning. First, recordings of the focus group sessions were listened to repeatedly by the first author. The first author made preliminary interpretations that were discussed with the last author. Second, the first author sorted the data guided by the preliminary interpretations and formed preliminary themes related to the purpose of the study. This meant that the sorted data was condensed to describe the content of the focus group discussions, and the meaning of the condensations was discussed in depth with the third author. Throughout this process, the themes and categories remained interconnected, allowing for continuous refinement. Ultimately, the goal was to create meaningful themes and categories and shed light on how they were interrelated, corresponding to the data’s underlying meaning.

## Results

### Dialogue as the foundation for structured and efficient care

The results provided support for the overall theme: “Dialogue as the foundation for structured and efficient care”. The introduction of the new way of working has turned the CFS into a valuable tool for structuring work based on the professionals’ different roles and for effectively communicating about patients’ needs. This new approach emphasizes collaboration at its core Frailty assessment serves as the starting point for dynamic dialogues that better address patient requirements. This is described in the first key theme, “Teamwork and dynamic dialogue”, with the sub-themes of “Frailty assessment – a joint picture” and “A person-centred approach – balance between patient and staff”, and the second key theme, “Efficiency – using resources correctly”, with the sub-theme “Active participation in team meetings – a prerequisite for efficiency” (see Table 1).

**Table 1:** Overview of findings

Overall theme	Dialogue as the foundation for structured and efficient care	
Key themes	***Teamwork and dynamic dialogue***	***Efficiency – using resources correctly***
Subthemes	*Frailty assessment – a joint picture*	*Active participation in team meetings – a prerequisite for efficiency*
	*A person-centred approach – balance between patient and staff*	

### Teamwork and dynamic dialogue

The new way of working has strengthened communication and cooperation among all professional categories. The staff now listen to each other more and are getting to know each other better on a personal level, which has positively impacted the team dynamics. Ideas are openly discussed, and input comes from all sides. Roles are clearer, creating a more cohesive team centred around the patient. In meetings, there is no rivalry; everyone has a voice, and patients are seen as persons. Learning about each other’s skills, working methods, mindsets, and personalities facilitates cooperation, even when there are differing views. As a result, team security increases, reducing the risk of overlooking critical aspects. These theme are further explored in the following two sub-themes, “Frailty assessment – a joint picture” and “A person-centred approach – balance between patient and staff”.

### Focus groups dialogues to illustrate the key theme “teamwork and dynamic dialogue”

RESPONDENT 4: Since we meet more often, or it feels like we talk more frequently and perhaps in a different way, it becomes easier for us across professions to plan things together. “Okay, are you going to try mobilizing after lunch? Then I can provide pain relief at that time. Let me know if there are any changes.”

RESPONDENT 5: I think, especially in rehab… I might be speaking for physiotherapists, but I believe we now have a closer dialogue with the doctors than we did before, as we didn’t see you as often. It makes a difference….

RESPONDENT 3: Yes, I can say that it’s easier to work now because we collaborate much more. Meeting every day allows us to quickly get a comprehensive picture of improvements, deteriorations, or changes.

INTERVIEWER: So, the teamwork, which you’ve mentioned several times, has improved because you meet more often?

RESPONDENT 5: Regarding person-centred care, we aim for patients to achieve optimal functional ability and be medically stable before discharge. However, there’s more to do. For instance, when reporting to the next caregiver, we could use it more to understand the patient’s own wishes and needs. It’s about better information transfer.

RESPONDENT 1: There’s a section called “Patient’s Own Expectations”, or what is it called…?

RESPONDENT 5: Yes, exactly, which we might not fully utilize.

RESPONDENT 1: Yes, it’s there actually.

### Frailty assessment – a joint picture

The frailty assessment serves as a point of departure for teamwork. It involves joint daily rounds where the entire team discusses and constructs an overall picture of the patient using a person-centred approach. Starting with the patient’s self-assessment of their abilities, the staff gain insight into their previous state. Often, the patients whom the staff encounter are the frailest, meaning that they typically are those who are the most physically weak and vulnerable. Fractures are merely symptoms of frailty, and when combined with other illnesses, patients become increasingly dependent on assistance for everyday activities. This patient group can deteriorate rapidly, necessitating flexible planning. The focus is on what the patient has managed previously and what they should aim for in the future. Enhanced communication and active participation in meetings allow everyone’s voice to be heard, creating a clearer structure for recovery and planning for the future.

### A person-centred approach – balance between patient and staff

A person-centred approach means recognizing the patient as more than their illness. Professionals collaborate with patients, combining their expertise. However, there can be discrepancies between this approach and organizational constraints. Patient wishes may clash with practical limitations faced by staff. Sometimes, patients and staff have divergent views on discharge requirements. Trusting the patient’s communication – even when it differs from staff assessments – is crucial. Achieving a balance between patient needs and operational realities is essential.

#### Focus groups dialogues to illustrate the subtheme a person-centred approach – balance between patient and staff

RESPONDENT 3: Sometimes, there can be a clash with the patient’s expectations, especially when discussing discharge. Patients often have a different understanding of what is required to be discharged from the hospital.

RESPONDENT 2: Nowadays, patients start asking about their discharge the day after surgery. I wish I could provide more person-centred care, but our frameworks limit our flexibility.

RESPONDENT 3: It’s important to communicate with patients based on person-centredness. Most patients are reasonable and understand the situation if their expectations are managed. Sometimes, patients refuse care or interventions because they think it will prevent them from being discharged. It’s crucial to involve patients early in their post-surgery process to help them understand and participate in their rehabilitation.

RESPONDENT 4: It’s challenging. Initially, patients may be confused, but by day three, they start to understand their situation better.

RESPONDENT 3: Yes, and then they might get an extra day.

RESPONDENT 4: Exactly.

### Efficiency – using resources correctly

This theme focuses on optimizing care by using resources efficiently. Effective teamwork enables staff to make the best use of available resources. Having a shared plan allows the team to consistently refer to it, minimizing unnecessary discussions. This approach fosters an objective and cohesive process involving everyone in the team. Clearly defined responsibilities for each professional category within the team enhance precision and rigour in the care process. Staff know precisely what tasks each professional should perform, which tests are necessary, and what information needs to be gathered. Patients also benefit from this clarity of responsibility, as they can promptly assess improvements or deteriorations in care. This theme is further explored in the sub-theme below.

### Active participation in team meetings: a prerequisite for efficiency

The working method promotes participation and active involvement. The staff are of the opinion that this leads to better care for their patients. Achieving this requires preparation and each professional being ready for the team round. Even when some of the staff are on leave or absent, it is important that everyone is familiar with and committed to the way of working. According to the staff, the following principles are important; by adhering to them, they can work efficiently and deliver optimal patient care.

#### Preparation

Before the round, the staff should be well-informed and complete the digital overview board. This ensures up-to-date information for effective decision-making. The digital overview board provides structure, complementing the information in the journal.

#### Exclusion and avoidance of duplication

Considering what can be excluded and avoiding unnecessary repetition makes it possible to work more efficiently.

#### Maintaining a positive atmosphere

Striving for a positive group atmosphere ensures that structure and assessment do not negatively impact relationships with each other or with patients.

#### Openness to feedback

Everyone, especially newcomers, should be receptive to giving and receiving suggestions and feedback.

#### Flat hierarchy

Rather than pointing fingers, the staff take responsibility for their respective areas. Feedback flows freely, regardless of experience.

### Focus groups dialogues to illustrate the subtheme “active participation in team meetings: A prerequisite for efficiency”

RESPONDENT 4: It is important that everyone is well-informed, especially during the CGA rounds. We need to collect as much information as possible for accurate assessments of our patients.

RESPONDENT 5: I liked the idea of actively contributing. It’s not just about attending the rounds and listening; everyone should contribute.

RESPONDENT 4: Exactly, everyone should contribute.

RESPONDENT 5: It’s just as much my responsibility as anyone else’s.

RESPONDENT 4: That’s very true.

RESPONDENT 5: Yes, actively participating is key.

RESPONDENT 4: Yes, actively participating is important.

RESPONDENT 5: Absolutely. When everyone participates, it works perfectly.

RESPONDENT 4: Speaking from the perspective of an assistant nurse, it’s not always easy. But in geriatrics, it feels easier because everyone’s role is valued. In our team, everyone contributes and is involved, and many assistant nurses feel truly involved.

## Discussion

The results show that staff perceive CGA to be working much better when frailty assessments use the CFS as a point of departure for dynamic dialogue, which in this study has been highly successful. Our study highlights the importance of having a joint picture, such as the one generated by the CFS, to gather around. In a recent systematic review, healthcare professionals’ contributions to interprofessional collaboration were examined [18]. The authors identified three key ways in which healthcare professionals collaborate: bridging gaps, negotiating roles and tasks, and creating space. Our study supports this by showing that healthcare professionals effectively negotiate these gaps, creating an environment where everyone benefits from each other’s skills. It is as if they are building bridges across professional divides, leading to dynamic dialogues. By using the frailty assessment as a common ground for gathering patient information, they speak the same language – whether they are nurses, nurse assistants, physicians, physical therapist, or occupational therapists. Staff members feel that this new approach provides more efficient care, optimizing resource utilization and creating a more precise and stringent care process. Research supports the use of the CFS to improve communication and coordination within healthcare teams [19–22]. This helps different care providers – from physicians to nurses and occupational therapists – to “speak the same language” when it comes to assessing and managing patients’ frailty. This, in turn, can lead to more efficient and coordinated care. However, to our knowledge, there are no studies that used teams as a tool to collectively conduct a frailty assessment using the CFS.

While the CFS undoubtedly is important, it is its combination with effective teamwork in CGA that has yielded positive outcomes. Interprofessional teamwork is defined as an intervention involving various health and social care professionals who share a team identity, work closely together, and collaborate in an integrated and interdependent manner to solve problems and deliver services [23, 24]. However, despite its effectiveness when carried out by interprofessional teams, it does not automatically lead to collaboration when professionals gather in such a team [25]. Communication often remains fragmented, resulting in separate efforts rather than cohesive interprofessional care [26]. To enable quality, safe, and accessible healthcare, interprofessional collaboration is essential. Research indicates that a limited understanding of each other’s roles and responsibilities can impact the approach of interprofessional teams [27]. Our study underscores the transformative potential of adopting innovative working methods, which can elucidate staff roles and cultivate a more unified team that places the patient at the heart of its collaborative efforts.

Our study also emphasizes the importance of working in a person-centred way and seeing each person as unique, even though there may be a discrepancy between the person-centred approach and what is feasible within the organization. Finding a balance between these two perspectives is crucial for providing the best person-centred care possible for patients. Person-centred care involves recognizing all persons as unique, with their own abilities and resources, and acknowledging their history and context as well as their strengths and weaknesses [28]. It also involves building trusting relationships between healthcare professionals and those in need of care. To achieve this, each person’s knowledge and experience must be considered when making joint decisions in partnership between older adults and staff. In the person-centred care approach, partnership also means shared decision-making at every step of the care process [4]. Caregivers should actively listen to patients’ stories and involve them in care planning and implementation [4]. By creating a partnership between patients and healthcare providers, individual needs can be balanced with organizational requirements. The study suggests that the patient group can deteriorate rapidly, necessitating flexible planning. The focus is on what the patient has managed previously and what they should aim for in the future. Enhanced communication and active participation in meetings allow everyone’s voice to be heard, creating a clearer structure for recovery and planning for the future.

Efficient resource utilization in healthcare – driven by staff members’ positive experiences with new working methods, as shown in this study – has significant benefits for society. This efficiency is crucial globally, as shown by a literature review [29], and specifically in Sweden, where maximizing health outcomes with public funds is a constant need. The staff’s positive experiences can drive healthcare improvement, as highlighted in a recent article on the NHS [21]. The article underscores the importance of intentional workforce management, emphasizing diversity, inclusion, and equity, and collaboration with staff, patients, and the public. In Sweden, as elsewhere, how healthcare staff’s experience their work is key to care quality. If staff feel effective, this can enhance patient care and staff satisfaction, contributing to cost-effective healthcare. This challenges the traditional top-down approaches, segmentation, and silo thinking prevalent in the Swedish healthcare system. In summary, positive staff experiences with new approaches drive healthcare improvement, benefiting patients and society. This impact stems from co-creation and collaborative development [30] among all stakeholders, including patients and those working close to the patients.

### Method discussion

Focus groups necessitate careful group composition to avoid strong uniformity [13]. Therefore, we made efforts to gather participants with shared experiences that varied in nature, considering both heterogeneity and homogeneity. What brought our participants together (homogeneity) were their shared experiences, which are crucial for stimulating discussion. On the other hand, heterogeneity, marked by differences between group participants, contributes to diversity within the chosen target group [15]. While efforts were made to balance homogeneity and heterogeneity, achieving the perfect mix is challenging. The shared experiences (homogeneity) were crucial for stimulating discussion, but the diversity (heterogeneity) within the group might not have been fully representative.

Previous research has shown that being grouped with others with the same experiences, being able to discuss things with people who understand, and knowing that you are not the only one with a particular experience all create a feeling of sharing [15]. The participants in this study seemed to appreciate the opportunity to take part in the focus groups, resulting in fruitful discussions in which the participants shared their views – both positive and negative. Negative views have been found to be expressed more easily in the presence of other participants who have something in common [13, 15]. The outcome of the discussions heavily depended on the involvement of the participants. In smaller groups, the dynamics can vary significantly based on individual engagement, which might have influenced the results. While negative views were expressed more easily in the presence of participants with similar experiences, this might have led to a bias where negative feedback was more prominent, potentially overshadowing positive aspects.

Most recommendations in the literature are for larger focus groups, with up to 12 participants [14]. According to this recommendation, each of the focus groups in the present study had a rather low number of participants. In this study, we planned for six participants in each group, but the actual number was five to six participants. Small groups of three to six participants have been shown to be very dynamic, and the outcome of the discussion depends more on the involvement of the participants than on their number [14, 15]. The awareness of sharing similar experiences can make participants realize that their views are legitimate and valid [13–15], which was the case in the present study. The study had smaller focus groups (five to six participants) compared to the recommended larger groups of up to 12 participants. Although smaller groups can be dynamic, the limited number of participants might have restricted the range of perspectives and the depth of discussion. However, the discussions in the groups were very vivid, so we do not believe this affected the study’s results.

## Conclusion

This study draws attention to the power of frailty assessment using the CFS as an essential part of CGA and as a shared tool for collaboration in the healthcare team. It promotes dynamic discussions, bridges professional divides, and allows healthcare professionals to effectively define roles and tasks. However, our study uniquely points out the lack of research exploring the team-based use of tools for conducting a frailty assessment using the CFS. This identifies new research opportunities to explore the potential advantages of such a collaborative approach in frailty assessment, which could further improve care efficiency and coordination, ultimately enhancing patient outcomes.

The research also underscores the criticality of efficient teamwork. It proposes that despite inherent obstacles, interprofessional teamwork can result in integrated care when a new working method is implemented that clarifies staff roles and focuses the team on the patient. The research also emphasizes the necessity of a person-centred approach, acknowledging everyone’s uniqueness and balancing personal needs with organizational demands. However, the research recognizes that the patient group can rapidly deteriorate, which requires adaptable planning.

In Sweden, as elsewhere, how the healthcare staff’s experience their work is crucial to the quality of care. When staff feel effective, this can improve patient care and staff satisfaction, contributing to cost-effective healthcare. This challenges the traditional top-down approaches, segmentation, and silo thinking prevalent in the healthcare system. The study asserts that positive staff experiences with new approaches drive healthcare improvement, benefiting patients and society. This underscores the potential for further improvements in healthcare delivery through continued innovation and collaboration.

## Data Availability

The datasets generated and analysed during the current study are not publicly available due to the information provided to the involved persons when obtaining their informed consent, stating that all attempts would be made to maintain their confidentiality. De-identified data are available upon reasonable request to enable review and will be stored for 10 years from publication at the University of Gothenburg, Sweden. All data are covered by the Swedish Public Access to Information and Secrecy Act (offentlighets- och sekretesslagen) and a confidentiality assessment (sekretessprövning) will be performed at each individual request. Permission from the University of Gothenburg, the Institute of Neuroscience and Physiology, must be obtained before data can be accessed. Contact synneve.dahlin-ivanoff@gu.se or dataskydd@gu.se for any requests.

## Abbreviations

CGA: Comprehensive Geriatric Assessment
CFS: Clinical Frailty Scale
